# Adjunctive duration-doubled transcranial direct current stimulation for the treatment of depressive patients with suicidal ideation: study protocol for a double-blind, randomized, sham-controlled trial

**DOI:** 10.1186/s13063-023-07858-0

**Published:** 2024-01-02

**Authors:** Yiming Chen, Dongbin Lyu, Fan Wang, Qinte Huang, Weichieh Yang, Mengke Zhang, Zheyi Wei, Shuxiang Shi, Shuqi Kong, Shentse Chen, Shuang He, Vivien Yang, Yiru Fang, Abdel Douiri, Wu Hong

**Affiliations:** 1 Department of Psychiatry & Affective Disorders Center, Ruijin Hospital, Shanghai Jiao Tong University School of Medicine, Shanghai, China; 2 Shanghai Mental Health Center, Shanghai Jiao Tong University School of Medicine, Shanghai, China; 3 Fuzhou Neuro-Psychiatric Hospital, Fujian, China; 4 Shanghai Key Laboratory of Psychotic Disorders, Shanghai, China; 5 CAS Center for Excellence in Brain Science and Intelligence Technology, Shanghai, China; 6 Hunan Second People’s Hospital (Hunan Brain Hospital), Hunan, China; 7 King’s College London, School of Life Course & Population Sciences, London, UK; 8 National Institute for Health Research Biomedical Research Centre (BRC), Guy’s and St Thomas’ NHS Foundation Trust and King’s College London, London, UK

**Keywords:** Transcranial direct current stimulation (tDCS), Major depressive disorder (MDD), Suicidal ideation, Randomized controlled trial (RCT)

## Abstract

**Background:**

The problem of suicide has become increasingly common in individuals with major depressive disorder (MDD). Transcranial direct current stimulation (tDCS) is an effective treatment for MDD with 2 milliamperes (mA) for at least 30 min per day for 2 weeks. This study aims to investigate the efficacy of daily duration-doubled tDCS as an adjunctive intervention for rapidly reducing suicidal ideation and improving depression in MDD patients.

**Methods:**

In this double-blind, randomized, sham-controlled study, 76 MDD patients with suicidal ideation are randomly assigned to either active (*n*=38) or sham (*n*=38) tDCS group. The anode and cathode are placed over the scalp areas corresponding to left and right dorsolateral prefrontal cortex (DLPFC), respectively, and each stimulation lasts for 60 min. The primary outcome is defined as change of Beck Scale for Suicide Ideation (BSI) after 5 and 10 sessions. The change of other clinical assessments, blood biomarkers related to suicidal ideation and depressive sumptoms are defined as secondary outcomes. Blood biomarkers related to suicidal ideation are collected at baseline and after 10 sessions.

**Discussion:**

This study suggests the adjunctive duration-doubled tDCS might be a novel method to rapidly reduce suicidal ideation and improve depressive symptom. The variation of biomarkers could be potential predictive models of suicide risk.

**Trial registration:**

The trial protocol is registered with ClinicalTrials.gov under protocol registration number NCT05555927. Registered on September 25, 2022.

**Supplementary Information:**

The online version contains supplementary material available at 10.1186/s13063-023-07858-0.

## Administrative information

This protocol was written in accordance with the Standard Protocol Items: Recommendations for clinical Interventional Studies (SPIRIT) guidelines [1]. Note: the numbers in curly brackets in this protocol refer to SPIRIT checklist item numbers. The order of the items has been modified to group similar items (see http://www.equatornetwork.org/reporting-guidelines/spirit-2013-statement-defining-standard-protocol-items-for-clinical-trials/).

**Table:** 

Title {1}	Adjunctive duration-doubled transcranial direct current stimulation for the treatment of depressive patients with suicidal ideation: study protocol for a double-blind, randomized, sham-controlled trial
Trial registration {2a and 2b}.	Please refer to Item 2a and registration via https://clinicaltrials.gov/ct2/show/NCT05555927 Date of registration: September 25, 2022
Protocol version {3}	3.0, June 27, 2023
Funding {4}	This study was supported financially by Research Proposal for the Capability Promotion Project for Research-Oriented Doctor at Shanghai Mental Health Center (2021-YJXYS-06). The materials including tDCS devices were supported by Shanghai “Science and Technology Innovation Action Plan” Medical Innovation Research (21Y11905600); Shanghai "Science and Technology Innovation Action Plan" Natural Science Foundation of Shanghai (21ZR1455100) and Davis Family Gift Fund. The professional service was supported by National Key R&D Program of China (2016YFC1307100); the National Natural Science Foundation of China (81930033); the Key Project of Clinical Research Center of Shanghai Mental Health Center (CRC2018ZD02); Shanghai Municipal Science and Technology Major Project (2018SHZDZX05); the Project of “Psychobehavioral Disorder” Clinical Research Center of Shanghai Jiao Tong University School of Medicine (2019-yxy-01) and Innovative Research Team of High-level Local Universities in Shanghai.
Author details {5a}	Yiming Chen^2^, Dongbin Lyu^2^, Fan Wang^2^, Qinte Huang^2^, Weichieh Yang^3^, Mengke Zhang^2^, Zheyi Wei^2^, Shuxiang Shi^2^, Shuqi Kong^2^, Shentse Chen^2^, Shuang He^2^, Vivien Yang^2^, Yiru Fang^1,2,4,5,6^, Abdel Douiri^7,8*^, Wu Hong^2,4*^ 1. Department of Psychiatry & Affective Disorders Center, Ruijin Hospital, Shanghai Jiao Ton University School of Medicine, Shanghai, China. 2. Shanghai Mental Health Center, Shanghai Jiao Tong University School of Medicine, Shanghai, China. 3. Fuzhou Neuro-Psychiatric Hospital, Fujian, China. 4. Shanghai Key Laboratory of Psychotic Disorders, Shanghai, China. 5. CAS Center for Excellence in Brain Science and Intelligence Technology, Shanghai, China. 6. Hunan Second People's Hospital (Hunan Brain Hospital), Hunan, China. 7. King's College London, School of Life Course & Population Sciences, London, United Kingdom. 8. National Institute for Health Research Biomedical Research Centre (BRC), Guy's and St Thomas' NHS Foundation Trust and King's College London, London, United Kingdom.
Name and contact information for the trial sponsor {5b}	Shanghai Mental Health Center. N 600 Wanping Nan Road, District Xuhui, Shanghai. Phone: 021-34773231.
Role of sponsor {5c}	The role of sponsors is to ensure that proper arrangements are in place to initiate, manage and report on this study.

## Introduction

### Background and rationale {6a}

Depression is a common mental disorer. Globally, it is estimated by the World Health Organization that 5% of adults suffer from depression. During the last decade, the problem of suicide has become more serious in individuals with major depression disorder (MDD). It is important to track whether alternative or adjunct treatment strategies have the potential to rapidly and effectively reduce suicide risk. Among brain stimulations, transcranial direct current stimulation (tDCS) has been proposed as a possible interventional tool. Over the past decade, trials [2, 3] and meta-analyses [4, 5] have shown mixed, albeit positive, results for tDCS as a treatment for MDD. However, trials or meta-analyses that analyzed the factor structure of depression, described as ‘suicide risk’, ‘suicide attempt’ or ‘suicidal ideation’ were rare.

On the other hand, until recently, the bulk of the literature into suicide-focused interventions centered around suicide attempts, particularly on preventing reattempt over the 6- to 12-month period after the index attempt [5]. In such trials, suicidal ideation is usually tracked as a secondary outcome measure. In contrast, evaluating rapid-acting interventions for suicidal ideation requires randomized clinical trials (RCTs) that focus on suicidal ideation as a primary outcome. Therefore, we highlight potential changes that could be made to clinical trials for suicidal ideation in the specific areas of participant selection, outcome measurement, and serum mechanisms of change.

Next, tDCS could be an effective method on depressive symptoms with a current of 2mA, 30 min per daily session and a therapeutic period of at least 2 weeks [6]. Near-linear effects of stimulation intensity (0.2 to 2.0 mA) and duration (from 1 to 13 min) were found on physiological effects of tDCS stimulation in motor cortex [7]. Since these early studies [7, 8], there has been a tendency in tDCS research to study the effects of increased duration or intensity of stimulation with the expectation of increased efficacy in behavioral and neurophysiological outcome measures. What is more, the excitement surrounding noninvasive neurostimulation as an adjunctive rapid-acting antidepressant with antisuicidal effects presents an important opportunity for framing studies in suicide research. In addition to offering an opportunity to evaluate the effects of tDCS as an adjunctive rapid-acting intervention on suicidal ideation and suicide risk, such initiatives could help researchers explore potential biomarkers of response and, ultimately, develop better treatments.

What is more, in recent years, a wide range of possible biological risk factors for suicide have also been proposed. As some recent trials demonstrated, systematic inflammation, indicated by increased levels of white blood cell (WBC) count or serum C-reactive protein (CRP), was also associated with later risk of suicide [9, 10]. Two studies even suggested that neutrophil-to-lymphocyte ratio (NLR) may be a marker for suicide vulnerability in patients with MDD [11, 12]. NLR was substantially higher in suicidal depressed patients compared with nonsuicidal depressed patients and healthy controls. One logistic regression model included NLR and previous attempts as predictive variables for suicide status [11]. What is more, NLR, monocyte-to-lymphocyte ratio (MLR), platelet-to-lymphocyte ratio (PLR), nerve growth factor (NGF), brain-derived neurotrophic factor (BDNF), glial cellline-derived neurotrophic factor (GDNF), interleukin (IL), soluble tumor necrosis factor receptor I (sTNFr1), and soluble tumor necrosis factor receptor II (sTNFr2) have been found to be associated with MDD [12, 13] as compared with healthy controls [14]. However, we are still far from having good biomarkers for either suicidal ideation or suicide attempt. Moreover, these possible biological risk factors were no more further investigated during or after certain antisuicidal treatment. And the possible association of their variation and suicide status change was not yet reported.

Therefore, any rapid-acting strategy for suicidal ideation has the potential to lead to new developments in clinical trial and to generate valuable clinical and research considerations for the field as a whole. Daily high-dose repetitive transcranial magnetic stimulation (rTMS) could be a novel and safe method for rapidly improving suicidal ideation in treatment-naive patients with MDD [15]. While most tDCS researches have focused on its antidepressive mechanisms, the field of preventing suicide is expanding to encompass important psychological considerations related to tDCS use that warrant discussion, including the best way to measure changes in suicidal ideation and the treatment of the suicidal individual. The present study will investigate the effects of daily duration-doubled tDCS as an adjunctive intervention for rapidly improving suicidal ideation and depression in MDD patients with antidepressants.

### Objectives {7}

The primary objective of this study is to investigate the acute efficacy on suicidal ideation, measured by the Beck scale for Suicidal Ideation (BSI) score. We hypothesize that a higher efficacy and larger reduction in suicidal ideation will be observed in tDCS group than sham group after 1 and 2 weeks when tDCS performed as an adjunctive treatment.

The secondary objectives include:

1. To investigate the acute improvement on other clinical measurements, measured by the 17-item Hamilton Depression Rating Scale (HAMD-17) score, etc., of duration-doubled tDCS or sham tDCS, after 1 and 2 weeks.
2. To investigate the potential association among variation of WBC, CRP, NLR, MLR, PLR, NGF, BDNF, GDNF, IL, TNF-α, sTNFr1, sTNFr2 and suicidal ideation as well as depressive symptoms in MDD patients [11–13] treated by adjunctive tDCS.
3. To investigate the clinical relevance of tDCS efficacy and side effect occurence.

### Trial design {8}

**Fig. 1:**
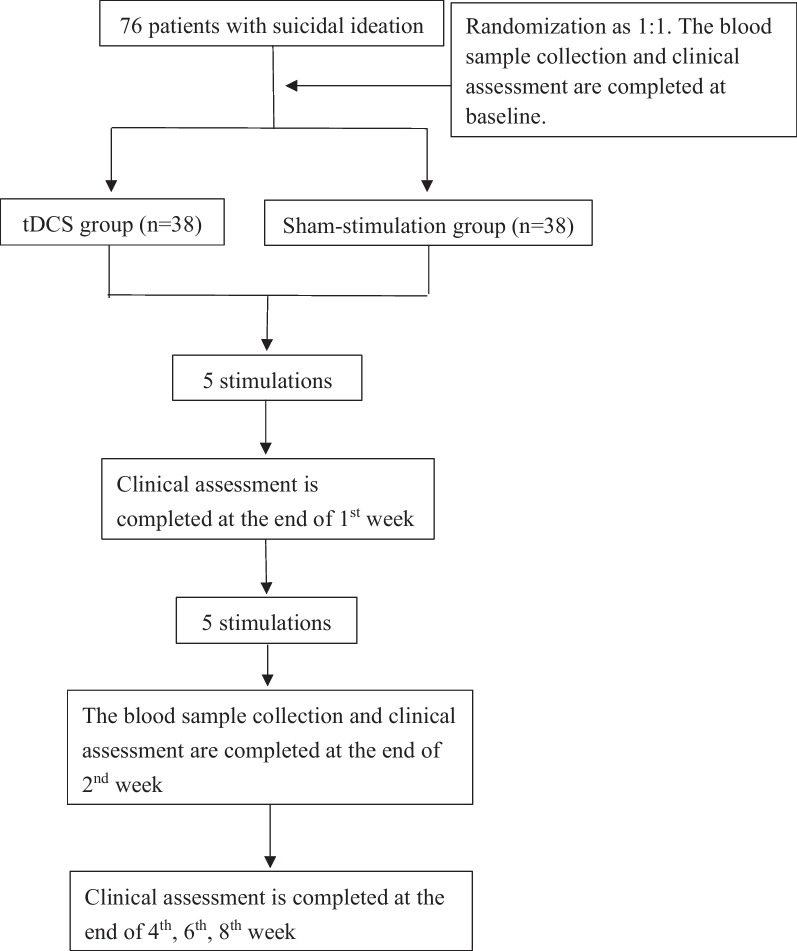
Trial flowchart. We plan to recruit 76 patients with MDD. For the patients who take no psychotropic drug at baseline or maintain actual pharmacotherapy for at least 2 weeks before the stimulation initiation and during the whole stimulation period. The patients will be randomized (1:1) into one of the following two groups: tDCS group or sham group. Primary and secondary study parameters will be accessed at baseline (week 0), the 1st week (after 5 sessions), 2nd week (after 10 sessions), 4th week (2 weeks after intervention), 6th week (4 weeks after intervention), and 8th week (6 weeks after intervention)

The design of the trial is a double-blinded, randomized, sham-controlled trial to test for the superiority of the active tDCS group versus sham group. MDD patients, who took no psychotropic drug at baseline or maintained actual pharmacotherapy for at least 2 weeks before the stimulation initiation and during the whole stimulation period, were included. Groups will be randomized through a random code generator software (REDCap) at ratio of 1:1 into one of the following two groups: tDCS group or sham group. The total study duration is 8 weeks (Fig. 1).

## Methods: participants, interventions, and outcomes

### Study settings {9}

We will approach patients at the Shanghai Mental Health Center outpatient and inpatient units by advertisement.

### Recruitment {15}

We will approach patients at the Shanghai Mental Health Center outpatient and inpatient units. Our inpatient and outpatients’ care unit follows at least 10,000 patients with MMD each year. Considering the inclusion and exclusion criteria, we expect approaching 100 patients to recruit 76 patients. Usual clinical care will be maintained for all patients following completion of the trial at our care unit. A well-trained group is involved in the participant enrolment process, including one senior psychiatrist, one PhD student, one MD student, eight master students working full-time, and ten psychiatrists who refer patients for scientific research. We provide the participants and their guardians with a thorough explanation to ensure that they fully understand the study.

Recruitment, pre-participation screening, allocation, intervention, and data collection will occur between September 2022 and March 2024. We plan that the study will be concluded, and the results will be available for analysis at the end of 2024. The present study protocol complies with the SPIRIT 2013 recommendations (Standard Protocol Items: Recommendations for International Trials) [1] (see Additional file 1). Figure 1 shows the schedule of enrollment, interventions, and assessments.

### Eligibility criteria {10}

#### Inclusion criteria

1. Ages are from 18 to 50;
2. Meeting the criteria of the Diagnostic and Statistical Manual of Mental Disorder (DSM-5) for current unipolar MDD, which is assessed by at least one professional psychiatrist;
3. Han ethnicity;
4. Right handedness;
5. With a score≥17 on the HAMD-17 [16];
6. With a score≥6 on the BSI [17];
7. Without any pharmacotherapy at baseline or maintaining actual pharmacotherapy for at least 2 weeks before the stimulation initiation and during the total stimulation period.

#### Exclusion criteria

1. Assessed through applying the Mini-International Neuropsychiatric Interview (MINI) [18] by professional psychiatrists that diagnosed any other current or past psychiatric axis-I disorders (except MDD in the patients);
2. Severe liver and kidney diseases, active endocrine diseases or clinical symptoms. Severe cardiovascular disease, respiratory system disease, haematologic diseases, and cancer;
3. Any clinically significant abnormal laboratory examination that may influence the health of participants;
4. A history of any significant medical illness such as neurological disorders (such as cerebral trauma and seizure disorder);
5. Known current psychosis as determined by the DSM-5 or a history of a non-mood psychotic disorder;
6. Current alcohol and drug abuse;
7. Current or planned pregnancy and/or lactation during the trial;
8. Abnormal scalp such as open wounds;
9. HAMD-17 item 3 (suicide) score=4;
10. Receiving modified electroconvulsive therapy (MECT) or rTMS in the past 1 month;
11. Participation in another clinical trial concurrently or no more than 1 month prior to randomization.

### Who will take informed consent? {26a}

The assessors will obtain written consent from all participants.

### Additional consent provisions for collection and use of participant data and biological specimens {26b}

On the consent form, the patients can decide to accept being collected plasma to test biomarkers and being contacted in the future regarding the results of the biomarkers. Patients who decline this option still participate in the study.

## Interventions

### Background and rationale: choice of comparators {6b}

Sham group has been chosen as the comparison group. See below item 11a.

### Intervention description {11a}

Participants will receive one of two interventions: tDCS group or sham group. For brain stimulation, tDCS-CT devices (Neuroelectrics, Starstim 8, USA) are used. The stimulation protocol A or B will be administered in a software matched by tDCS device and by a staff member outside the research group with password. The research group cannot obtain the password during the trial. The participants will receive tDCS stimulation of protocol A or B as randomized from start to finish. The anode and cathode electrodes are inserted in saline-soaked sponges with diameter of 3.2cm and then positioned over the left and right dorsolateral prefrontal cortex (DLPFC) using specific headgear [19]. Each session uses a 2mA current and lasts 60 min. Ten sessions are daily performed on weekday. For sham, the current rapidly ramp up to 2mA over the first 30s and rapidly ramped down to 0mA over the next 30s automatically to allow participants to feel typical initial sensations of active tDCS [20, 21]. Trained psychiatrists and psychologists blind to group assignment administered the tDCS regimen. After completing ten sessions, no treatment is interdictory.

### Criteria for discontinuing or modifying allocated interventions {11b}

1. If participants do not meet the inclusion criteria;
2. If participants or their guardians decide to leave the study;
3. If participants develop a serious physical disease whether or not it is related to tDCS;
4. If participants stop taking their medication for three consecutive days, stop effective contraception or become pregnant;
5. If participants use other treatments (including MECT, rTMS);
6. If other conditions occur, and the investigator decides to terminate the study;
7. Defaulting.

During 10 sessions, modification of dose is not permitted. When ceasing treatment, no treatment is interdictory and participants could follow psychiatric routine.

### Strategies to improve adherence to interventions {11c}

During the intervention, every session is completed by tDCS-CT devices (Neuroelectrics, Starstim 8, USA) conducted by Neuroelectrics’ software according to the result of randomization as group A or B. Sessions are recorded in one document, including starting and ending time, name of operator, adverse effect of each session. Process of each session was performed as the same.

### Interventions: concomitant care {11d}

The participants in the study are not permitted to participate in other trials during the 2-week’s interventions. Most patients will receive concomitant individual pharmacotherapeutic treatment during this 2-week’s interventions.

### Provisions for post-trial care {30}

No provisions for post-trial care are planned. Patients could follow psychiatric routine after the completion of 10 sessions.

### Outcomes {12}

#### Primary outcome

The primary outcome is defined as reductive ratio of BSI after 1 and 2 weeks, as well as, after 5 and 10 sessions.

#### Secondary outcomes

The secondary outcome measures include change in other clinical assessments, such as HAMD-17 (range 0-54), Montgomery–Asberg Depression Rating Scale (MADRS, range 0-60), Oxford Depression Questionnaire (ODQ, range 0–104), Young Mania Rating Scale (YMRS, range 0–60), Clinical Global Impression Scale (CGI, severity range 0–8; efficacy range 1–4; efficacy index range 0–16), Sheehan Disability Scale (SDS, range 1–90), Wisconsin card sorting test (WCST), and Stroop color–word test (SCWT) after 5 and 10 sessions. And biomarkers representing potential mechanisms associated with suicidal ideation and depressive symptoms, after 5 and 10 sessions. What is more, the clinical relevance of these chosen efficacy and side effects occurence is also included as one of the secondary outcomes.

### Sample size {14}

The sample size was calculated based on the data from Kalu et al. [22] and Boggio et al. [23]. In these studies, the active tDCS was assumed to be superior to sham tDCS in controlling depression with a conservative mean diference of 0.74 and standard deviation (SD)=25.8. We set the type 1 error, *α* (significance), and the a power at 80%. We used G power software (V.3.1.9.4) to calculate the sample size. The result indicated that a minimum of 30 patients per group are needed (a total of 60 patients). Considering the 20% drop-out rate, 75 patients are needed in our experiment. Finally, we planned to enrol 38 patients per group for a total of 76 included patients.

## Assignment of interventions: allocation

### Sequence generation {16a}

Patients are randomly assigned to either of two groups—the active tDCS group or the sham tDCS group based on a computer-generated randomization scheme (REDCap) operated by the study staff, signed as group A or B. REDCap is an electronic data capture tool. The randomization program is set up by SC. When the baseline assessment is completed, their contact information is sent to the allocation team that will assign the participants to either group A or B. The allocation is randomized and computer generated. The randomization cannot be influenced by the person making it or any other person. Participants were not able to accurately guess what treatment they would receive. Clinician scales are administered by a trained and blinded study staff to ensure consistency. Moreover, we also checked the internal consistency for all psychiatrists involved in scale ratings with a kappa value of 0.89.

Two senior psychiatrists experienced with the MINI scale guarantee an accurate diagnosis. All patients who fulfil the inclusion criteria will be randomized. Randomization will be requested by the psychiatrists who are responsible for recruitment and clinical interview.

### Concealment mechanism {16b}

Personnel who are not blind to the treatment arm are responsible for the randomization process where REDCap is used. When a participant has been recruited for the study and baseline assessment has been conducted, the person responsible informs the assessor the result of randomization, group A or B, process via email. The actual intervention will be unknown to the researchers and clinicians. The randomized intervention allocation is concealed until the statistical analyses of the resulting data have been completed and conclusions have been drawn.

### Implementation {16c}

Randomization is carried out by a staff member outside the research group who generates the allocation sequence and assigns participants to interventions.

## Blinding

### Who will be blinded {17a}

Participants, care provider, investigator, and outcomes assessor are blind to the information about group allocation.

### Procedure for unblinding if needed {17b}

The participants are instructed in advance not to reveal allocation to researchers in any evaluation. We do not anticipate any requirement for unblinding but if required, the trial manager and data coordinator will have access to group allocations and any unblinding will be reported. Principal investigator (PI, YC) will feed data into the computer.

## Data collection methods

### Plans for assessment and collection of outcomes {18a}

#### Primary study parameter/endpoint

##### BSI

The primary outcome criterion is the reductive ratio in BSI score after 1 and 2 weeks of the treatment. A treatment response is defined as a 50% reduction in the BSI score and remission is defined as score of 6 in the BSI at any point during the 8-week trial compared with the baseline.

Secondary study parameters

1. Mini-International Neuropsychiatric Interview (MINI);
2. HAMD-17, MADRS, and ODQ;
3. YMRS;
4. CGI;
5. SDS;
6. WCST and SCWT;
7. Peripheral venous blood: Biomarkers representing inflammatory mechanisms associated with suicidal ideation and MDD are assessed. (1) Routine tests: The routine tests include calculation of the WBC, CRP, neutrophils, lymphocytes, monocytes, platelets. (2) Specific tests: We collect the peripheral blood serum and store it at −80°C after centrifugation. We examine the NGF, BDNF, GDNF, IL, TNF-α, sTNFr1, sTNFr2. NLR, MLR, and PLR in serum using ELISA.

Data collection will be performed by staff of study who will be trained in how to do assessments. Participants will be familiarized with the equipment and tests of study during screening. For all participants, data collection will be conducted at a certain time, in face-to-face meetings. The peripheral blood collection will be done at the laboratory in Shanghai Mental Health Center.

Participants will receive a SMS text or an email to remind their appointment the day before. PI (YC) of the study will supervise data collection to check the quality, completeness, plausibility, and possible errors of the collected data.

### Plans to promote participant retention and complete follow-up {18b}

Combined with a pragmatic approach, and an emphasis on the importance of participating in research, this hopefully keeps enrolled participants in the study and makes them complete follow-up interviews. Outcome data will also be collected for participants who discontinue or deviate from intervention protocols.

### Participant timeline {13}

All the time points for the physical examinations, scale assessments, medication use inventories and laboratory examinations are shown in Table 1.

**Table 1:**
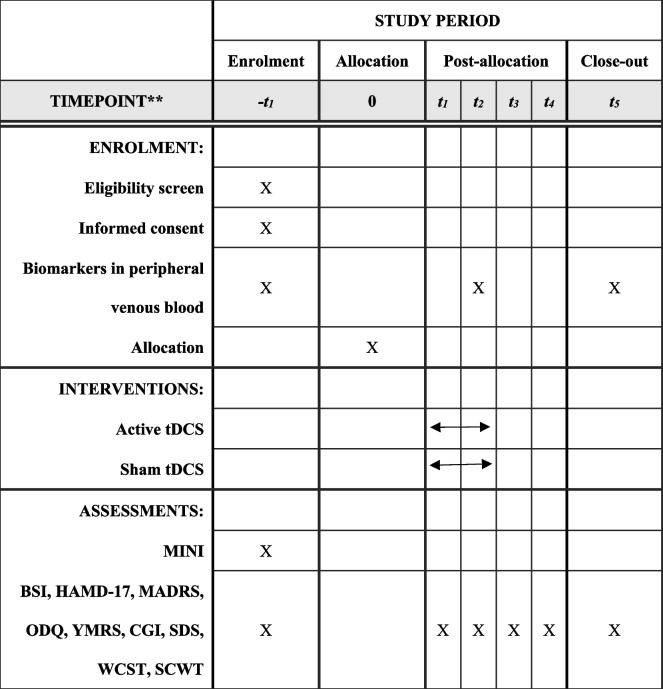
Data collection methods and clinical assessment time points

**−*t***_***1***_: 2 weeks before allocation; ***t***_***1***_: the 1st week (after 5 sessions); ***t***_***2***_: the 2nd week (after 10 sessions); ***t***_***3***_: the 4th week (2 weeks after intervention); ***t***_***4***_: the 6th week (4 weeks after intervention); ***t***_***5***_: the 8th week (6 weeks after intervention)

### Data management {19}

At the beginning of the study, participants will be assigned a unique identification code and the data sent to the study assessor will only include the code without participant identification information. Subjective assessments and demographic information collected on paper forms first and then will be entered into specific SPSS files by PI. And a second study staff member will check the data entry process for accuracy. All paper-based data for the study including consent forms, identifiable information, objective questionnaires, and subjective assessments will be kept separate from each other in a locked cabinet in the PI’s office of Shanghai Mental Health Center. The electronic records of the study, i.e., raw data of biomarkers, and the SPSS files stored on a password-protected external hard drive and computer. Both paper and digital documents will only be accessible to the research team. All of the data management work will be done at Shanghai Mental Health Center. A complete back-up of the primary database will be performed twice a month via netdisk with password.

### Composition of the data monitoring committee, its role and reporting structure {21a}

A Data Monitoring Committee (DMC) has been established in the hospital to monitor study conduct, the safety, efficacy. The DMC is composed of clinicians independent of the study and statistician and independent from the sponsor and competing interests.

### Interim analyses {21b}

We perform an interim analysis on the primary endpoint when 30 and 70% of patients have been randomized and have completed the 8-week follow-up. The interim analysis is performed by an independent statistician. The statistician will report independently to the DMC. The research team will have access to these interim results and the DMC could make the final decision to continue or terminate the trial.

### Confidentiality {27}

All study-related information and participant information will be stored in locked file cabinets in areas with limited access. All laboratory specimens and data reports will be accessed by a coded ID number. All records that contain names or other personal identifiers will be stored separately from study records. All local databases will be accessed by password. PI will be given access to the cleaned data sets. All data will be password protected. To ensure confidentiality, project team members will be blinded to the data.

### Plans for collection, laboratory evaluation and storage of biological specimens for genetic or molecular analysis in this trial/future use {33}

The total amount of blood collected per patient at each visit was 20ml. Blood samples are collected from peripheral veins and are obtained by standard techniques. They will be tested in laboratory of Shanghai Mental Health Center. The subject’s number and visit number should be pre-labeled on each specimen tube, at which time the researcher will add the patient’s personal information to the label. Biological specimens should be exhausted or disposed after statistical analysis. Storage of biological specimens for genetic or molecular analysis for future use in ancillary studies is not intended.

## Statistical methods

### Statistical methods for primary and secondary outcomes {20a}

Descriptive analysis will consist of means and SD for continuous variables and percentages for categorical variables. Data normality will be tested with the results expressed as mean ± SD or percentage, whenever appropriate. Non-parametric tests will be used if data were not normally distributed. Baseline variables will be compared between the groups using either the Student *t* test or chi-squared test (e.g., sociodemographic data, cognitive function data, biomarkers representing inflammatory mechanisms associated with suicidal ideation and MDD, response and remission rates between groups). To compare primary endpoint, a two-way ANOVA will be used considering group (intervention and sham-control groups) and time points (pre and post-intervention), followed by the Bonferroni post hoc test, in case of significant F ratios. Using a mixed-effects model for repeated measures to analyze change from baseline of BSI scores, time, and their interaction. We will also adopt pretreatment values (e.g., age, gender, baseline BSI, HAMD-17, CRP, and blood cells level) as covariates to correct the possible differences. An intention-to-treat analysis will be conducted, i.e., all participants, regardless of adherence to the intervention, will be included in the final analysis. All calculations will be performed using the R-softawre, and the significance level will be set at *P* ≤ 0.05.

### Methods for additional analyses {20b}

No additional analyses are planned for the study.

### Methods in analysis to handle protocol non-adherence and any statistical methods to handle missing data {20c}

Missing data are analyzed according to the intention-to-treat principles, i.e., analyzing individuals to their allocated groups regardless of, e.g., protocol non-adherence. Multiple imputations will be used to handle missing data.

### Plans to give access to the full protocol, participant-level data, and statistical code {31c}

The datasets analyzed during the current study and statistical code are available from the corresponding author on reasonable request, as is the full protocol.

### Composition of the coordinating center and trial steering committee {5d}

The Trial Steering Committee (TSC), which consists of representatives from the initiating and participating institutions, is the principal policy and decision-making committee of the trial and is responsible for the scientific conduct of the study.

### Responsibilities of TSC

YF, AD, and WH review the progress of study and necessary changes to the protocol to facilitate the smooth running of the study. The TSC will also ensure that the trial is conducted in accordance with the relevant principles and will provide overall supervision of the trial. All aspects of local organization (including recruitment, identifying potential recruits, obtaining consent, providing day to day support for the trial and medical responsibility of the patients) will be provided by the PI and YC. And the research coordinators (DL, FW, QH, WY, MZ, ZW, SS, SK, SC, SH, VY) will operate tDCS device, collect data, and follow-up the patients. The PI and the research coordinators meet weekly to oversee conduct and progress.

### Harms {22}

We record the possible side effects for each participant. Our trained team reports these data throughout the study using the serious adverse event reporting system. It is unlikely that the participants will be affected by a serious adverse event (SAEs) or a serious adverse reaction in this trial. Common adverse reactions include dizziness, headache, and scalp tingling. Only unexpected serious adverse events / reactions which are unrelated to these clinical procedures will be reported as SAEs. Additionally, Shanghai Mental Health Center has the insurance to cover all non-negligent harm associated with the protocol.

### Auditing {23}

The Ethics committee of Shanghai Mental Health Center at Shanghai Jiao Tong University will continue to review the trial. The TSC will check consent forms, compliance with the protocol and the planned tDCS interventions, and the quality of data collected in the case report forms at least annually.

### Plans for communicating important protocol amendments to relevant parties {25}

All changes will be communicated to sponsors and other relevant parties. In addition, deviations from the published protocol will be documented in the trial registration on ClinicalTrials.gov (Unique Protocol ID: 2021-YJXYS-06). Important amendments will be communicated to participants and reconsent obtained where appropriate.

## Patient public involvement

No public or patient involvement in the design of the protocol.

### Dissemination plans {31a}

Incidences from negligence (e.g., major protocol violations) will not be covered by study insurance. The PI will review all publications and be considered for lead author derived from this study. No later than 5 years after the ending of recruitment, we will deliver the whole deidentified data set to an appropriate data archive. All the results obtained from the present research will be published in some articles.

## Discussion

In this double-blinded, randomized, sham-controlled trial, we aim to determine the acute effectiveness of duration-doubled tDCS on suicidal ideation in patients with MDD. In addition to their usual treatment, participants will be randomly assigned to receive either 10 weekday sessions of active (2 mA) or sham tDCS as an adjunctive treatment, with the anode over the left DLPFC and the cathode over the right DLPFC. We will regularly assess suicidal ideation, depression severity, and functional impact using the BSI, HAMD-17, MADRS, ODQ, YMRS, CGI, and SDS throughout the trial. We will assess cognitive changes using WCST and SCWT, and also regularly assess treatment-related side effects using validated scales.

As suicidal ideation is regarded as one of the most important component in depression, the study will examine how suicidal ideation and depression change in participants with MDD throughout the trial period. Entrance and exit interviews will be recorded and analyzed by staffs in this research team in Shanghai Mental Health Center.

The study has numerous strengths and will follow the CONSORT (Consolidated Standards of Reporting Trials) 2010 statement on design and reporting [24]. Using REDCap to randomly assign participants to treatment groups will ensure that allocation is truly concealed, documented, and unchangeable. To minimize the placebo effect, both participants and treating investigators will be blinded. Primary and secondary outcomes were chosen to capture a range of subjective, objective, and functional measurements of suicidal ideation, depression severity, and the regular use of adverse event scales will ensure that treatment side effects are also adequately captured. Measurements will also be taken at multiple points during and after the trial to better describe the overall course and persistence of any treatment-related effects.

This study is unique for several reasons. It is the first of its kind to examine a MDD population with suicidal ideation, in which participants will have suffered from this mental disorder. The use of a relatively intensive tDCS regimen, in terms of session duration, is also unique among studies conducted with MDD. To our knowledge, this is also the first tDCS study for MDD that includes detailed measures of suicidal ideation. This may be useful in revealing the effect of this duration-doubled tDCS on suicidal ideation and in building a database of anonymized recordings for use in future analyses.

This study also has several practical limitations that may affect the results. With regard to treatment modality, delivering tDCS in a hospital setting will impose a minimal burden on inpatients, but outpatients will have to be functional enough to arrange for transport to the hospital on a daily basis. As such, there is a risk that the study population may miss outpatients with severe depression. The need for daily travel to the hospital can also pose problems with participant compliance. We may use home-based tDCS in future studies to mitigate this, although funding limitations prevented us from employing this approach in this trial. Second, our blinding procedure follows that used in most other studies of tDCS, but recent evidence suggests that this may not sufficiently mitigate the placebo effect. Turi et al. [25] showed that healthy volunteers were able to distinguish the fade-in, short stimulation, fade-out method of sham stimulation, described above, from true active stimulation at a level significantly greater than chance. It is not yet known how generalizable these findings are to a population with depression, but there is a need for more research into alternative methods of blinding in tDCS studies.

## Conclusions

In conclusion, this trial is a pragmatic, randomized, double-blinded, and sham-controlled study that seeks to compare the impact of active versus sham duration-doubled tDCS in treating MDD patients with suicidal ideation. To our knowledge, this study is the first of its kind in this patient population and also the first to conjointly employ detailed measures of suicidal ideation, which will also be used in future analyses. Given the immense health, economic, and societal impacts of MDD, new and effective treatments beyond pharmacotherapy and psychotherapy are needed, and tDCS may prove useful in this regard.

### Trial status

This trial is in the enrolment stage. The protocol was registered with ClinicalTrials.gov under protocol registration number NCT05555927 on September 25, 2022. Recruitment began on October 10, 2022. Recruitment is expected to be completed in March 2024.

### Supplementary Information

**Additional file 1.** SPIRIT 2013 Checklist: Recommended items to address in a clinical trial protocol and related documents*.

## Abbreviations

BDNF: Brain-derived neurotrophic factor
BSI: Beck Scale for Suicide Ideation
CGI: Clinical Global Impression Scale
CONSORT: Consolidated Standards of Reporting Trials
CRP: C-reactive protein
DLPFC: Dorsolateral prefrontal cortex
DMC: Data Monitoring Committee
DSM-5: Diagnostic and Statistical Manual of Mental Disorders, Fifth Edition
GDNF: Glial-cell line derived neurotrophic factor
HAMD-17: 17-item Hamilton Depression Rating Scale
MADRS: Montgomery–Asberg Depression Rating Scale
MECT: Modified electroconvulsive therapy
MLR: Monocyte-to-lymphocyte ratio
IL: Interleukins
MDD: Major depressive disorder
MINI: Mini-International Neuropsychiatric Interview
NGF: Nerve growth factor
NLR: Neutrophil-to-lymphocyte ratio
ODQ: Oxford Depression Questionnaire
PI: Principal investigator
PLR: Platelet-to-lymphocyte ratio
RCTs: Randomized clinical trials
rTMS: Repetitive transcranial magnetic stimulation
sTNFr1: Soluble tumor necrosis factor receptor 1
sTNFr2: Soluble tumor necrosis factor receptor 2
t_1_: After 5 sessions
t_2_: After 10 sessions
t_3_: At the end of 4 weeks
t_4_: At the end of 6 weeks
t_5_: At the end of 8 weeks
t_6_: At the end of 12 weeks
tDCS: Transcranial direct current stimulation
TNF-α: Tumor necrosis factor-alpha
TSC: Trial Steering Committee
SAEs: Serious adverse events
SCWT: Stroop color–word test
SD: Standart deviation
SDS: Sheehan Disability Scale
WBC: White blood cell
WCST: Wisconsin card sorting test
YMRS: Young Mania Rating Scale
